# Supplementary material for: Systematic mapping of antibiotic cross-resistance and collateral sensitivity with chemical genetics
Source: Nat Microbiol. 2024 Dec 2;10(1):202–16. doi: 10.1038/s41564-024-01857-w (PMC11726442; doi:10.1038/s41564-024-01857-w)
Supplement: Supplementary file 1 — Reporting Summary [file 41564_2024_1857_MOESM1_ESM.pdf]

nature portfolio

Corresponding author(s): Dr. Athanasios Typas (typas@embl.de)

Last updated by author(s): Oct 10, 2024

Reporting Summary

Nature Portfolio wishes to improve the reproducibility of the work that we publish. This form provides structure for consistency and transparency in reporting. For further information on Nature Portfolio policies, see our Editorial Policies and the Editorial Policy Checklist.

Statistics

For all statistical analyses, confirm that the following items are present in the figure legend, table legend, main text, or Methods section.

n/a	Confirmed	
☐	☒	The exact sample size (*n*) for each experimental group/condition, given as a discrete number and unit of measurement
☐	☒	A statement on whether measurements were taken from distinct samples or whether the same sample was measured repeatedly
☐	☒	The statistical test(s) used AND whether they are one- or two-sided<br>*Only common tests should be described solely by name; describe more complex techniques in the Methods section.*
☒	☐	A description of all covariates tested
☐	☒	A description of any assumptions or corrections, such as tests of normality and adjustment for multiple comparisons
☐	☒	A full description of the statistical parameters including central tendency (e.g. means) or other basic estimates (e.g. regression coefficient) AND variation (e.g. standard deviation) or associated estimates of uncertainty (e.g. confidence intervals)
☐	☒	For null hypothesis testing, the test statistic (e.g. *F*, *t*, *r*) with confidence intervals, effect sizes, degrees of freedom and *P* value noted<br>*Give P values as exact values whenever suitable.*
☒	☐	For Bayesian analysis, information on the choice of priors and Markov chain Monte Carlo settings
☒	☐	For hierarchical and complex designs, identification of the appropriate level for tests and full reporting of outcomes
☐	☒	Estimates of effect sizes (e.g. Cohen's *d*, Pearson's *r*), indicating how they were calculated

Our web collection on statistics for biologists contains articles on many of the points above.

Software and code

Policy information about availability of computer code

Data collection	Biomek FX (Beckman Coulter); SoftMax Pro 7.1; BioTek Gen5 (v.3.02.2); TECAN i-control (v1.10);
Data analysis	R v.4.1.2; R packages drc (v0.5.8), Bioconductor (v3.16); Python (v3.9.17); Python package scikit-learn (v1.1.3), Python library SciPy (v1.12.0)

For manuscripts utilizing custom algorithms or software that are central to the research but not yet described in published literature, software must be made available to editors and reviewers. We strongly encourage code deposition in a community repository (e.g. GitHub). See the Nature Portfolio guidelines for submitting code & software for further information.

Data

Policy information about availability of data

All manuscripts must include a data availability statement. This statement should provide the following information, where applicable:

- Accession codes, unique identifiers, or web links for publicly available datasets
- A description of any restrictions on data availability
- For clinical datasets or third party data, please ensure that the statement adheres to our policy

All supplementary data is provided in supplementary tables. A reference genome from the NCBI database (E. coli BW25113 strain K-12 chromosome; GCF_000750555.1) was used. Raw reads of sequenced samples (file names are describe samples) are in Zenodo DOI: 10.5281/zenodo.10572857 All data is included in the Shiny app: https://shiny-portal.embl.de/shinyapps/app/21_xrcs

Research involving human participants, their data, or biological material

Policy information about studies with human participants or human data. See also policy information about sex, gender (identity/presentation), and sexual orientation and race, ethnicity and racism.

Reporting on sex and gender	N/A
Reporting on race, ethnicity, or other socially relevant groupings	N/A
Population characteristics	N/A
Recruitment	N/A
Ethics oversight	N/A

Note that full information on the approval of the study protocol must also be provided in the manuscript.

Field-specific reporting

Please select the one below that is the best fit for your research. If you are not sure, read the appropriate sections before making your selection.

☒ Life sciences ☐ Behavioural & social sciences ☐ Ecological, evolutionary & environmental sciences

For a reference copy of the document with all sections, see nature.com/documents/nr-reporting-summary-flat.pdf

Life sciences study design

All studies must disclose on these points even when the disclosure is negative.

Sample size	For all experiments, no prior assumptions were made regarding sample size. In the validation experiment (Fig. 3b-d) we used 12 lineages per antibiotic and 7 lineages in evolution against drug combinations (Fig. 6). Experiments were performed in at least two biological (independent overnight cultures) and maximum of six biological replicates (Fig. 5). Given the number of lineages and number of biological replicates per lineages, the sample size is sufficient for evolution experiments. For number of replicates for each experiment, please see figure legends and "Methods".
Data exclusions	No exclusions were performed for experimental data obtained in this project (for details please see "Methods").
Replication	Experiments were always conducted in at least two and at max six biological (independent overnight cultures). For number of replicates for each experiment, please see figure legends and "Methods".
Randomization	Randomization was not relevant to our study. Randomization is less relevant in experimental evolution of antibiotic resistance, the main experimental method of our study, because the primary focus is on how a specific selective pressure, like antibiotics, drives predictable evolutionary changes. Since bacterial populations are often clonal and mutations arise spontaneously, the outcomes are largely determined by this selective pressure rather than random variation. As a result, randomization of experimental conditions is unnecessary, as the experiment is designed to observe resistance development in response to a controlled environment. Controls were always processed in parallel.
Blinding	For in vitro experiments, no blinding was performed, since it was necessary to keep track of which samples are used in each experiments. Controls were always processed in parallel.

Reporting for specific materials, systems and methods

We require information from authors about some types of materials, experimental systems and methods used in many studies. Here, indicate whether each material, system or method listed is relevant to your study. If you are not sure if a list item applies to your research, read the appropriate section before selecting a response.

Materials & experimental systems

n/a	Involved in the study
☒	☐ Antibodies
☒	☐ Eukaryotic cell lines
☒	☐ Palaeontology and archaeology
☒	☐ Animals and other organisms
☒	☐ Clinical data
☒	☐ Dual use research of concern
☒	☐ Plants

Methods

n/a	Involved in the study
☒	☐ ChIP-seq
☒	☐ Flow cytometry
☒	☐ MRI-based neuroimaging

Plants

Seed stocks	N/A
Novel plant genotypes	N/A
Authentication	N/A
